# Endocarditis Complicated by Severe Aortic Insufficiency in a Patient with COVID-19: Diagnostic and Management Implications

**DOI:** 10.1155/2020/8844255

**Published:** 2020-09-29

**Authors:** David J. Sanders, Joanne S. Sutter, Antone Tatooles, Tisha M. Suboc, Anupama K. Rao

**Affiliations:** ^1^Department of Internal Medicine, Section of Cardiology, Rush University Medical Center, Chicago, IL, USA; ^2^Department of Cardiovascular and Thoracic Surgery, Rush University Medical Center, Chicago, IL, USA

## Abstract

A 38-year-old man presented with cough, shortness of breath, and fatigue. He was diagnosed with Coronavirus Disease-2019 (COVID-19) as well as *Enterococcus faecalis* bacteremia. Imaging revealed a subaortic membrane with aortic valve endocarditis and severe aortic insufficiency. He had successful aortic valve replacement with a mechanical prosthesis and subaortic membrane resection. This case highlights some of the diagnostic and therapeutic challenges presented by COVID-19 pandemic.

## 1. Introduction

The Coronavirus Disease-2019 (COVID-19) pandemic is an unparalleled global crisis that has commanded health resources and public attention. Although there is an overwhelming focus on this new disease, the emergence of SARS-CoV-2, COVID-19's causative organism, has not eliminated other maladies. Patients continue to have common etiologies for their presentation, and multiple illnesses can complicate and coexist with COVID-19. Additionally, concerns about the infectivity and potential aerosolization of SARS-CoV-2 impose barriers to performing invasive procedures on such patients.

Here, we present a case of aortic valve infective endocarditis (IE) complicated by severe aortic insufficiency (AI) in a patient simultaneously diagnosed with COVID-19. We illustrate how COVID-19 can have similar clinical characteristics to IE, among other cardiovascular diseases. We also highlight our thought process on the risks and benefits of transesophageal echocardiogram (TEE) and aortic valve replacement surgery in this patient and discuss the importance of individualized decision-making.

## 2. Case

A 38-year-old man with end-stage renal disease from hypertensive nephropathy requiring hemodialysis through a tunneled dialysis catheter presented with two weeks of nonproductive cough, shortness of breath, and fatigue. The patient's initial vital signs were notable for a blood pressure of 84/46, pulse of 83, respirations of 20, and oxygen saturation of 97% on room air. Though initially afebrile, he mounted a temperature of 101.5°F several hours after presentation. On admission, his physical exam was notable for a II/VI systolic ejection murmur, not previously documented.

A chest X-ray revealed pulmonary vascular congestion and a right retrocardiac opacity. ECG showed sinus rhythm, borderline 1^st^ degree AV block, and left anterior fascicular block. Laboratory evaluation demonstrated white blood cell count of 14.5 K/*μ*L, troponin of 0.1 ng/mL, BNP of 148 pg/mL, C-reactive protein of 108 mg/L, and ferritin of 1685 ng/mL. A nasopharyngeal swab detected SARS-CoV-2 RNA, confirming the diagnosis of COVID-19. Blood cultures were also obtained on admission, which grew *Enterococcus faecalis* from consecutive specimens.

**Figure 1:**
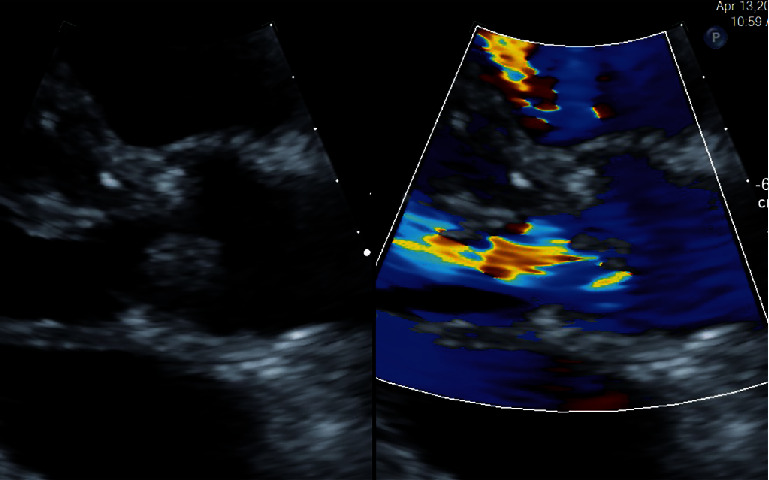
TTE from parasternal long axis view showing large echodensity on the right coronary cusp. Color Doppler demonstrates AI.

The patient was initiated on an IV antibiotic regimen of vancomycin and gentamicin. However, persistent bacteremia was noted despite several days of antibiotic therapy. His dialysis catheter was exchanged in case it was the infectious source. Given the murmur on exam and ongoing bacteremia, the patient underwent a transthoracic echocardiogram (TTE) for further evaluation. This revealed a 10 × 11 mm mobile echodensity on the right coronary cusp as well as a possible smaller vegetation on the left coronary cusp of the aortic valve. Additionally, there was eccentric aortic insufficiency (AI) graded as moderate in severity by qualitative assessment (Figure 1).

**Figure 2:**
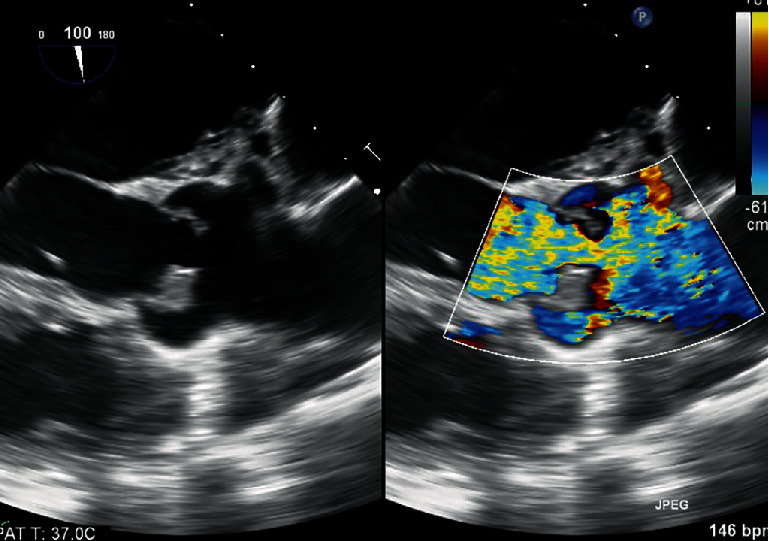
TEE from transgastric long axis view with beam path at 120 degrees showing aortic valve echodensity. Color Doppler demonstrates severe AI. Yellow arrow points to subaortic membrane.

To further characterize the aortic valve and assess for paravalvular abscess, a transesophageal echocardiogram (TEE) was then performed. All personnel donned appropriate personal protective equipment (PPE) including controlled air purifying respirators (CAPR). The TEE confirmed two aortic valve vegetations: (1) 8 × 14 mm on the right coronary cusp and (2) 3 × 4 mm on the left coronary cusp. There was nonspecific thickening of the posterior aortic root, raising concern for a paravalvular abscess. Multiple parameters suggested his AI was severe: jet width ratio > 65%, jet area ratio > 60%, vena contracta of 0.6, and premature closure of the mitral valve. Interestingly, a linear structure in the left ventricular outflow tract (LVOT) was visualized, suggestive of a subaortic membrane (Figure 2).

**Figure 3:**
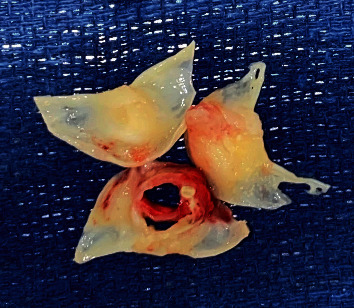
Resected aortic valve with vegetation on the left coronary cusp and perforation of the right coronary cusp.

**Figure 4:**
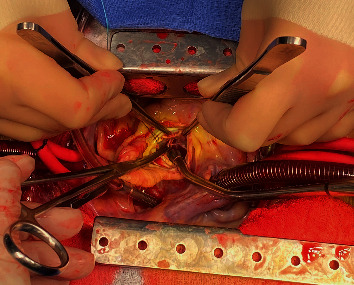
Intraoperative images showing the thin fibrotic subaortic membrane visible above the suction cannula.

A multispecialty team including cardiology, cardiovascular surgery, and infectious disease determined that despite his COVID-19, the patient required urgent aortic valve surgery given the severity of his AI, persistent bacteremia, vegetation size > 10 mm, and concerns for paravalvular abscess. The day following his TEE, the patient was taken to the operating room. He was found to have extensive phlegmon on the right coronary cusp and a perforation of the left coronary cusp of the aortic valve (Figure 3). Additionally, inspection of the LVOT confirmed a fibrotic subaortic membrane extending from the interventricular septum to the anterior annular region of the mitral valve (Figure 4). The native aortic valve was resected and replaced with a 25 mm St Jude mechanical prosthesis. The subaortic membrane was resected.

The patient tolerated the procedure well and did not have any postoperative complications. He was extubated a few hours following the surgery. His aortic valve culture grew *Enterococcus faecalis*. His SARS-CoV-2 PCR was also repeated and remained positive. The patient's antibiotics for IE were switched to ampicillin and ceftriaxone based on sensitivity data, and he was discharged home to complete a six-week course.

## 3. Discussion

As our case illustrates, the COVID-19 pandemic has presented new challenges for the diagnosis and treatment of cardiovascular disease. Patients with COVID-19 most commonly present with fever and cough and can have an array of nonspecific complaints [1]. There is a symptom overlap with other cardiovascular conditions such as acute coronary syndrome, decompensated heart failure, non-COVID-19 myocarditis, and IE. Clinicians have to remain vigilant to the possibility of these other illnesses, or they may be overlooked. Relying on heuristics, or mental shortcuts, in making a diagnosis could lead to delays and adverse outcomes [2]. In particular, the dominant focus on COVID-19 brings this diagnosis to mind more easily than others (availability bias) [2]. Attributing all of the given patient's symptoms to COVID-19 after a positive test (premature closure) may lead to other common prepandemic diagnoses, such as IE, being missed [2].

For our patient, it was not clear which disease process caused his symptoms: COVID-19 respiratory infection, bacterial IE with pulmonary congestion from AI, or some combination of the two conditions. Testing for multiple diagnoses from the beginning was critical for timely diagnosis.

The original source for the patient's bacteremia was suspected to be his tunneled dialysis catheter. Additionally, his previously undiagnosed subaortic membrane likely created an ideal substrate for aortic valve seeding. The patient's TTE identified a vegetation > 10 mm and associated AI, high-risk features that suggested a greater chance of a complicated course or need for surgery [3]. This earned a class I indication for urgent TEE to further assess for complications [3].

In this patient with COVID-19, it was especially necessary to carefully consider the risks and benefits of a TEE. TEEs are aerosol-generating procedures that could increase the risk of transmitting SARS-CoV-2 to those involved [4, 5]. To minimize staff exposure, computed tomography (CT) or magnetic resonance imaging (MRI) can serve as an alternative for some indications [6]. While multimodality imaging might be a useful adjunct for IE, CT and MRI may not have comparable spatial and temporal resolution to a TEE for the diagnosis of endocarditis [6]. Postponing this procedure would have delayed characterizing the intracardiac complications and determining the need for surgical intervention. Thus, in our case, TEE was deemed essential and performed utilizing proper personal protective equipment (PPE).

The TEE results produced a similar dilemma for surgical planning. The study confirmed large vegetation size, severe AI, and possible early aortic root abscess, all indications for early surgery, which is associated with reduced embolic events and mortality [3, 7, 8]. Additionally, the patient had persistent bacteremia despite being on appropriate antibiotic therapy. And, given the unpredictable course of acute severe AI and potential for abrupt clinical deterioration, the multispecialty team determined that delaying surgery would be too unsafe. Lastly, postponing surgery risked further valve destruction and, given the vegetation's location, progression to aortic root abscess formation and subsequent heart block.

Proceeding with surgery before the patient had cleared the COVID-19 infection did pose hazards to the surgical staff and required special precautions. This included minimizing the number of operating room personnel, using N95 masks and other PPE throughout surgery, and coordinated handling of equipment and specimens.

As the pandemic continues, we will have to continue to carefully weigh the risks of delaying care to the potential hazards of exposing clinical staff to the virus. Cases that are elective or time sensitive now have the potential to become urgent or emergent. As with this case, individualized decision-making may be key to providing timely and appropriate care.
